# DNA methylation and copy number variation profiling of T-cell lymphoblastic leukemia and lymphoma

**DOI:** 10.1038/s41408-020-0310-9

**Published:** 2020-04-28

**Authors:** Zahra Haider, Mattias Landfors, Irina Golovleva, Martin Erlanson, Kjeld Schmiegelow, Trond Flægstad, Jukka Kanerva, Ulrika Norén-Nyström, Magnus Hultdin, Sofie Degerman

**Affiliations:** 1 Department of Medical Biosciences, Umeå University, Umeå, Sweden; 2 Department of Radiation Sciences, Umeå University, Umeå, Sweden; 3 Department of Paediatrics and Adolescent Medicine, Rigshospitalet, Copenhagen University Hospital, Copenhagen, and Institute of Clinical Medicine, Faculty of Medicine, University of Copenhagen, Copenhagen, Denmark; 4 Department of Pediatrics, University of Tromsø and University Hospital of North Norway, Tromsø, Norway; 5 New Children’s Hospital, Helsinki University Hospital and University of Helsinki, Helsinki, Finland; 6 Department of Clinical Sciences, Umeå University, Umeå, Sweden; 7 Department of Clinical Microbiology, Umeå University, Umeå, Sweden

**Keywords:** Lymphoma, Leukaemia

## Abstract

Despite having common overlapping immunophenotypic and morphological features, T-cell lymphoblastic leukemia (T-ALL) and lymphoma (T-LBL) have distinct clinical manifestations, which may represent separate diseases. We investigated and compared the epigenetic and genetic landscape of adult and pediatric T-ALL (*n* = 77) and T-LBL (*n* = 15) patient samples by high-resolution genome-wide DNA methylation and Copy Number Variation (CNV) BeadChip arrays. DNA methylation profiling identified the presence of CpG island methylator phenotype (CIMP) subgroups within both pediatric and adult T-LBL and T-ALL. An epigenetic signature of 128 differentially methylated CpG sites was identified, that clustered T-LBL and T-ALL separately. The most significant differentially methylated gene loci included the *SGCE/PEG10* shared promoter region, previously implicated in lymphoid malignancies. CNV analysis confirmed overlapping recurrent aberrations between T-ALL and T-LBL, including 9p21.3 (*CDKN2A/CDKN2B*) deletions. A significantly higher frequency of chromosome 13q14.2 deletions was identified in T-LBL samples (36% in T-LBL vs. 0% in T-ALL). This deletion, encompassing the *RB1, MIR15A* and *MIR16-1* gene loci, has been reported as a recurrent deletion in B-cell malignancies. Our study reveals epigenetic and genetic markers that can distinguish between T-LBL and T-ALL, and deepen the understanding of the biology underlying the diverse disease localization.

## Introduction

T-cell acute lymphoblastic leukemia (T-ALL) and T-cell lymphoblastic lymphoma (T-LBL) are precursor lymphoid neoplasms, characterized by the uncontrolled proliferation of progenitor T-cells. A lymphoid neoplasm with a bone marrow infiltration of malignant blast cells by more than 20–25% is classified as T-ALL^1^. However, if the bone marrow infiltration is less than 25% and the primary disease localizes in the mediastinum, lymph nodes or the extramedullary tissues, it is classified as T-LBL^1^. Despite differences in clinical manifestation, T-ALL and T-LBL have an overlapping immunophenotype and recurrent genetic aberrations, such as *NOTCH1* activating mutations, *CDKN2A/B* deletions (cell cycle defects) and loss-of-heterozygosity in chromosome 6q^2,3^. Previous studies^4,5^ have identified gene expression differences between pediatric T-ALL and T-LBL, but no genetic or epigenetic markers has yet been defined to distinguish between the two malignancies, which are currently differentiated solely based on bone marrow infiltration. DNA methylation classification has been implicated as a prognostic and diagnostic marker in various cancers including hematological malignancies^6–8^. We have identified a CpG island methylator phenotype (CIMP) panel, consisting of 1293 specific CpG sites, which classified pediatric T-ALL patients into clinically relevant subgroups^6,7^. The CIMP− (low methylation) T-ALL patient subgroup had a significantly worse prognosis compared to the CIMP+ (high methylation) subgroup^6,7,9^.

The aim of this study was to further analyze DNA methylation-based heterogeneity in T-cell lymphoblastic malignancies, with focus on investigating if the previously reported CIMP subgroups identified in pediatric T-ALL were also present in T-LBL and adult T-ALL patients. Furthermore, the methylomic and genomic landscape of T-ALL and T-LBL were compared using high-resolution genome-wide DNA methylation and copy number variation (CNV) detection arrays to investigate and identify molecular markers that could distinguish between T-ALL and T-LBL. Such epigenetic and genetic markers can give an insight into the biological mechanisms underlying the divergent clinical manifestation of the two neoplasms, as well as reveal novel therapeutic targets and strategies.

## Materials and methods

### Patient samples

Bone marrow and fresh frozen lymph node tissue samples from diagnostic (*n* = 76) and late relapsed (*n* = 1) T-ALL and diagnostic (*n* = 15) T-LBL patients were retrieved along with complete remission bone marrow samples (*n* = 4). The adult (age > 18 years, *n* = 7) and pediatric (age ≤ 18 years, *n* = 8) T-LBL patients, adult T-ALL patients (age > 18 years, *n* = 12), were diagnosed at University Hospital of Umeå, Sweden, between years 1998 and 2012. The study included all available T-ALL and T-ALL samples diagnosed during the specified time period and no further selection was done. The diagnosis was based on morphologic, cytogenetic and immunophenotypic analysis, according to the WHO classification of lymphoid neoplasms^10^. Patients were classified as T-ALL or T-LBL based on blast cell infiltration in the bone marrow, according to previously described guidelines^1^. The 65 Nordic pediatric (age < 18 years) T-ALL patient samples have been previously described and analyzed in our lab by HumanMethylation450K arrays (data deposited to the NCBI Gene Expression Omnibus (GEO) repository accession no. GSE69954)^6^.

From the GEO repository, methylation profiles of normal immature (CD34+) and mature hematopoietic (CD3+) cells (GSE49618)^11^, and normal bone marrow (*n* = 7) and lymph node (*n* = 5) tissues (GSE50192)^12^, were retrieved. Gene expression data of pediatric T-ALL (*n* = 10) and T-LBL (*n* = 20) samples analyzed by Affymetrix HG-U133Plus2.0 GeneChip arrays were also retrieved from GEO (GSE29986)^5^.

The study was approved by the Regional and/or National Ethics Committees and the patients and/or their guardians provided informed consent in accordance with the Declaration of Helsinki.

### DNA Extraction and bisulfite conversion

DNA extracted from freshly frozen lymph nodes and bone marrow tissue samples were sodium bisulfite converted using Zymo EZ DNA methylation kit (Zymo Research, CA, USA) according to the manufacturer´s protocol.

### DNA Methylation array analysis, CIMP classification, and epigenetic and mitotic age prediction

Illumina´s HumanMethylation450K BeadChip arrays (Illumina Inc., San Diego, CA) were used for genome-wide methylation profiling of bisulfite converted DNA. These arrays interrogate 485,577 CpG sites across the genome and the average beta (avg. β) methylation level of each CpG site (CpGs) ranges from 0 (unmethylated) to 1 (fully methylated).

The raw methylation data was extracted using the Genome Studio software (Illumina), and the data was preprocessed, normalized and filtered, as described previously^6^ (Supplementary Fig. S1). The methylation data were corrected for the bias introduced by different bead types in the methylation array (BMIQ normalization)^13^, and batch-effect corrected using ChAMP Bioconductor package version 2.8.9^14^ with default parameters.

CIMP classification by the 1293 CpGs CIMP panel was carried out as previously described^6^. Briefly, patient samples with >40% methylated CpGs (avg. β > 0.4) in the CIMP panel were classified as CIMP high (CIMP+) whereas patients with ≤40% methylated CpGs were classified as CIMP low (CIMP−).

DNA methylation-based models were used to predict epigenetic and mitotic age^15,16^.

### Differential methylation analysis

Differential methylation analysis of promoter-associated CpGs i.e., CpG sites located up to 1500 base pair upstream of genes, (*n* = 146035 CpGs) was performed between T-ALL and T-LBL using ChAMP^14^ (Supplementary Fig. S1). Batch effect corrected methylation data were analyzed using default parameters and significant differentially methylated CpGs (DM-CpGs) with adjusted *p*-value < 0.05 and absolute delta beta ≥0.3 were retained (Supplementary Fig. S1). Functional analysis was performed using the functional annotation clustering tool in Database for Annotation, Visualization and Integrated Discovery (DAVID) v6.7^17^. Clusters including more than 10 genes and with an enrichment score >1.0 were selected as significant (Supplementary Table S1).

### CNV analysis by DNA methylation arrays

The raw signal intensity data from the HumanMethylation450K arrays was imported to R by the minfi package^18^ and CNV analysis was performed by the conumee package^19^, where two T-ALL complete remission bone marrow samples were used as reference. Parameters and limits for deletion and gains were set for each individual array through manual inspection. The CNV analysis included four complete remission samples as negative controls in order to identify possible false positives due to technical biases or germline variation. CNVs identified by the segmentation with fewer than 10 observations, as defined by the conumee package, were excluded from the analysis. CNV segments overlapping in three or more samples in either T-ALL or T-LBL and in fewer than three of the four negative controls were summarized with the minimal common region. One of the samples was excluded as the limits could not be set with any certainty due to high variation. Group comparisons of CNVs were performed using Fisher’s exact test on individual CNVs. Human genome GRCh37 (NCBI)/hg19 (UCSC) was used for assigning all chromosome positions.

### CNV analysis by genome-wide SNP array

Genome-wide copy number variation (CNV) analysis was performed using whole-genome single-nucleotide polymorphism (SNP) Infinium CytoSNP-850K v1.1 BeadChip microarrays (Illumina), covering approximately 850 000 SNPs. In accordance with the manufacturers’ protocol, 200 ng DNA was hybridized on a beadchip after whole-genome amplification, after which the arrays were scanned using the HiScan instrument (Illumina). Genotyping results were visualized, normalized and clustered using Genotyping module of the GenomeStudio software (Illumina). The cnvPartition 3.2.0 (Illumina) was applied for CNV detection by retrieving Log R Ratio (LRR, the ratio between the observed and the expected probe intensity) and the B Allele Frequency (BAF). Deviations from the expected values indicate copy number alterations. Human genome GRCh37 (NCBI)/hg19 (UCSC) was used for assigning all chromosome positions.

### Bioinformatic and statistical analysis

Principal component analysis (PCA) of centrally scaled DNA methylation (avg. β values) and log2 scaled gene expression (average signal values) data was carried out using SIMCA version 14 (Umetrics, Umeå, Sweden). For statistical analysis, the Statistical Package for the Social Sciences (SPSS Inc., Chicago, IL) software version 24 was used along with R.

Statistical tests included Fisher´s exact test for comparing categorical variables and independent samples *T*-test for comparing continuous variables. Welch´s two sample *T*-test was used to compare log2 transformed gene expression data. Cluster analyses were performed using Wards method with Euclidean distance metric.

## Results

### Demographic data

A total of 15 T-LBL (7 adult and 8 pediatric), 77 T-ALL (12 adult and 65 pediatric) patient samples, and four complete remission samples were analyzed using DNA methylation arrays. Publicly available DNA methylation array data of normal bone marrow and lymph node samples^12^, as well as sorted CD3+ and CD34+ cells^11^ were further included as reference samples. There were no significant differences in age and gender distribution between T-ALL and T-LBL patients, and both malignancies showed higher prevalence in males (Table 1).

**Table 1:** Demographic data of T-ALL and T-LBL study cohort.

	T-ALL		T-LBL		T-ALL vs. T-LBL (adult/pediatric) *p*-values
	Adult	Pediatric	Adult	Pediatric	
No. of samples	12	65	7	8	–
Male/female (% male)	8/4 (66.7%)	43/22 (66.2%)	5/2 (71.4%)	7/1 (87.5%)	1/0.42^a^
Age, years (median, range)	35 (21–65)	7 (1–17)	33 (19–77)	10.5 (3–18)	0.91/0.34^b^
CIMP status, +/− (% CIMP+)	8/4 (66.7%)	40/25 (61.5%)	5/2 (71.4%)	5/3 (62.5%)	1/1^a^

^a^Fisher´s exact test, ^b^Independent samples *T*-test.

The T-ALL and T-LBL patient samples were CIMP classified according to the previously defined CIMP panel, consisting of 1293 CpGs^6^. Irrespective of disease (T-ALL vs. T-LBL) and age group, the frequency of CIMP+ was similar (61.5–71.4%) (Table 1).

### Genome-wide DNA methylation profiling

**Fig. 1:**
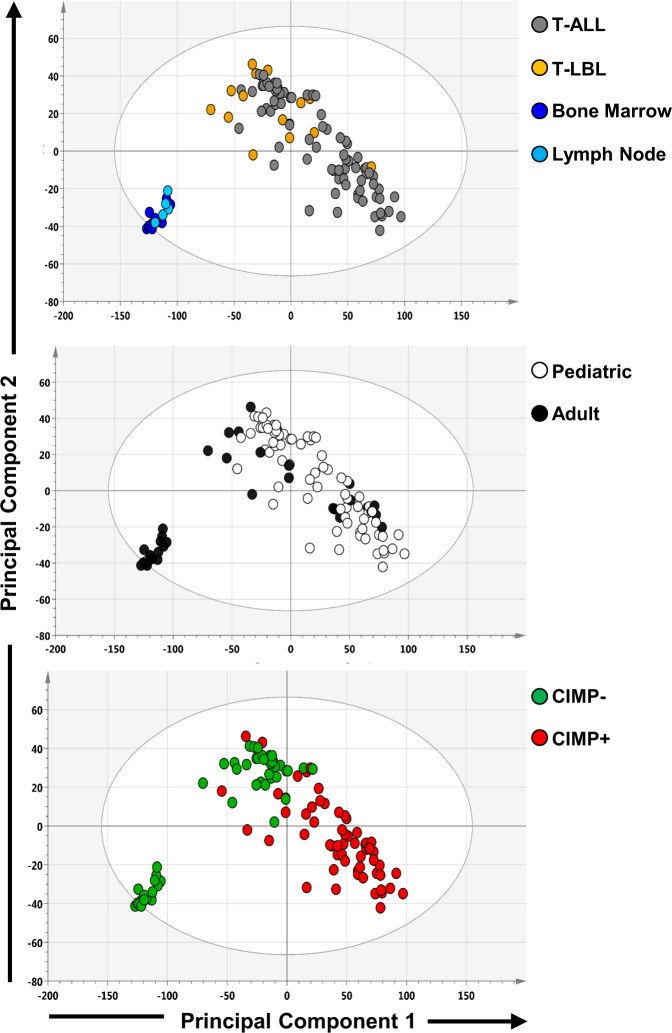
Principal component analysis (PCA) of global DNA methylation. PCA scoring plots based on the average β values of 397 316 CpGs. The samples in the PCA plots are colored by (**a**) sample type: T-ALL (*n* = 77), T-LBL (*n* = 15), and non-malignant lymph node (*n* = 5) and bone marrow (*N* = 11) reference tissue, (**b**) Age group: pediatric (*n* = 73) and adult (*n* = 19) and (**c**) CIMP status (59 CIMP+ and 33 CIMP−).

Genome-wide DNA methylation patterns across adult and pediatric T-ALL and T-LBL patient samples along with reference samples were explored by principal component analysis (PCA) analysis on batch-effect corrected DNA methylation array data (Fig. 1, Supplementary Fig. S2). The PCA plots were generated using center scaled beta (avg. β) values of the genome-wide CpGs retained after filtering (*n* = 397316), comprising of 76% gene-related and 24% intergenic CpGs. The PCA did not project T-ALL and T-LBL as separate clusters, nor on patients’ age group in the first and second principal components (Fig. 1). However, the non-malignant reference samples (bone marrow and lymph node origin) clustered separately from the T-ALL and T-LBL samples, reaffirming large methylomic variation between normal and malignant tissues. Notably, CIMP status separated the malignant samples into CIMP+ enriched cluster and CIMP− enriched cluster. This shows the presence of CIMP subgroups in both T-ALL and T-LBL (Fig. 1). Similar clustering was observed upon performing PCA using only promoter-associated CpG sites (Supplementary Fig. S2).

### CIMP subgroups have differential predicted cellular age

**Fig. 2:**
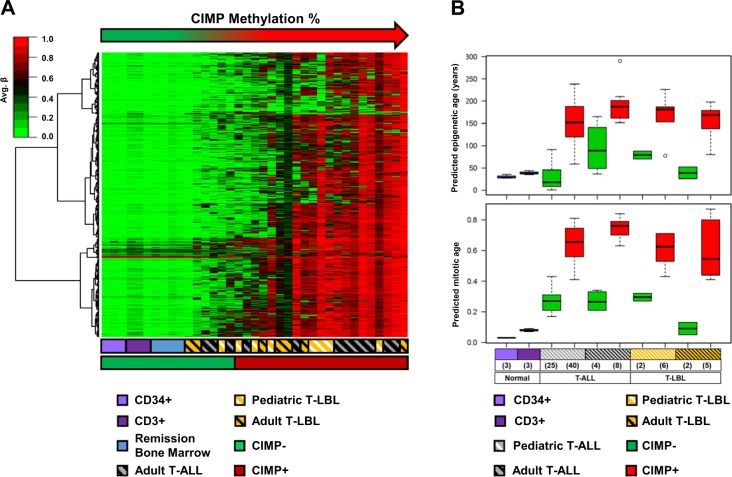
Validation of CIMP subgroups in T-ALL and T-LBL. **a** The heat map shows average β values of 1293 CpGs (as rows) in the CIMP panel of T-ALL and T-LBL samples along with reference samples including tumor-free bone marrow remission samples (*n* = 4) and sorted CD34+ (*n* = 3) and CD3+ (*n* = 3) cells. The samples (columns) are sorted according to increasing CIMP methylation percentage. **b** Epigenetic and mitotic age, predicted using Horvath and Yang et al. models^15,16^, compared between CIMP subgroups in T-ALL and T-LBL.

Adult T-ALL samples and pediatric/adult T-LBL samples were CIMP classified based on the CIMP panel identified in pediatric T-ALL^6^. This analysis confirmed the presence of CIMP subgroups in all age groups of T-LBL and T-ALL (Fig. 2a).

The CIMP subgroups in T-LBL and adult T-ALL had similar characteristics as the CIMP subgroups in pediatric T-ALL^9^ (Fig. 2a). The methylation profile of the CIMP− malignant samples were closer to the CIMP profile of the normal reference samples (complete remission samples and normal sorted CD34+ and CD3+ T-cells), whereas the CIMP+ samples were more hypermethylated compared to the reference samples (Fig. 2a).

The CIMP subgroups in pediatric T-ALL have been previously shown to have differential replicative histories^9^, reflected by significant differences in predicted epigenetic and mitotic age^9,15,16^. Similar differences in epigenetic age and mitotic age were shown in the CIMP subgroups within T-LBL and adult T-ALL, with the CIMP+ samples having an older predicted epigenetic^15^ and mitotic age^16^ (Fig. 2b).

### Differential DNA methylation analysis between T-ALL and T-LBL

**Fig. 3:**
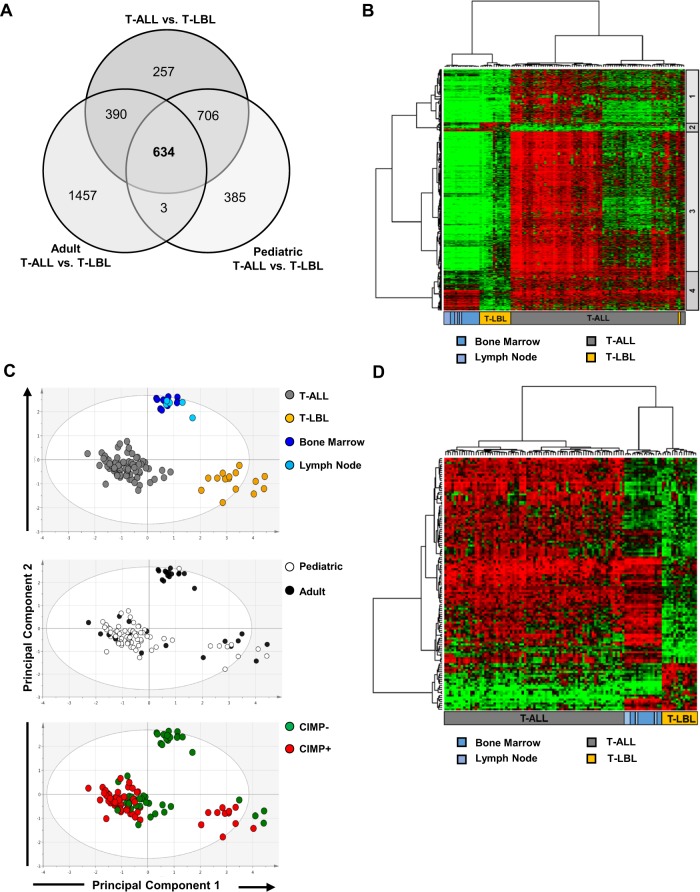
Differential DNA methylation analysis between T-ALL and T-LBL. **a** Venn diagram showing the common, overlapping 634 DM-CpGs between T-ALL and T-LBL. **b** Heat map of avg.β values of the 634 DM-CpGs in T-ALL, T-LBL and non-malignant reference samples. The hierarchical cluster analysis of the DM-CpGs generated four clusters of CpG sites which are marked on the right. **c** Heat map showing 128 DM-CpGs from cluster 2 and 4 (from Fig. 3b), that represent methylomic differences between T-ALL and T-LBL, independent of CIMP profiles. **d** PCA of the 128 DM-CpGs showing the separation of T-ALL and T-LBL samples, based on tissue type, age group and CIMP status.

Despite the overlapping global DNA methylation patterns, T-ALL and T-LBL have different patterns of disease distribution and clinical manifestation. In order to investigate methylomic differences between T-ALL and T-LBL, differential methylation analysis was performed using the ChAMP algorithm focusing on promoter-associated CpG sites (*n* = 146,035). To avoid identifying age-related DNA methylation differences, differential methylation analysis was carried out firstly, between all T-ALL (*n* = 77) and T-LBL (*n* = 15) samples, secondly, between adult T-ALL (*n* = 12) and adult T-LBL (*n* = 7) and lastly, between pediatric T-ALL (*n* = 65) and pediatric T-LBL (*n* = 8) (Supplementary Fig. S1, Supplementary Table S2). A total of 634 differentially methylated CpGs (DM-CpGs), with an adjusted *p*-value < 0.05 and absolute delta β (absΔβ) value ≥ 0.3, were common between all three analyses (Fig. 3a–b).

Hierarchical clustering separated the 634 DM-CpGs and analysis was focused on the two clusters (cluster 2 and 4) that were not associated with CIMP class (Fig. 3b, Supplementary Fig. S3). Cluster 2, consisting of 24 DM-CpGs hypermethylated in T-LBL and cluster 4, with 104 DM-CpGs hypomethylated in T-LBL were identified as DM-CpGs with the most variable methylation profile between T-ALL and T-LBL samples, and not associated with CIMP profiles (Fig. 3c, Supplementary Fig. S3). PCA using avg. β values of the 128 DM-CpGs (from clusters 2 and 4) clearly separated T-ALL and T-LBL samples, without discriminating between different patient age groups or the CIMP phenotype (Fig. 3d). Furthermore, the methylation signature of the 128 DM-CpGs did not separate the non-malignant lymph nodes and bone marrow reference samples, showing that the DM-CpGs did not represent tissue-specific methylation differences between T-ALL and T-LBL (Fig. 3d).

### Functional relevance of DM-CpGs between T-ALL and T-LBL evaluated by integrated gene expression analysis

**Fig. 4:**
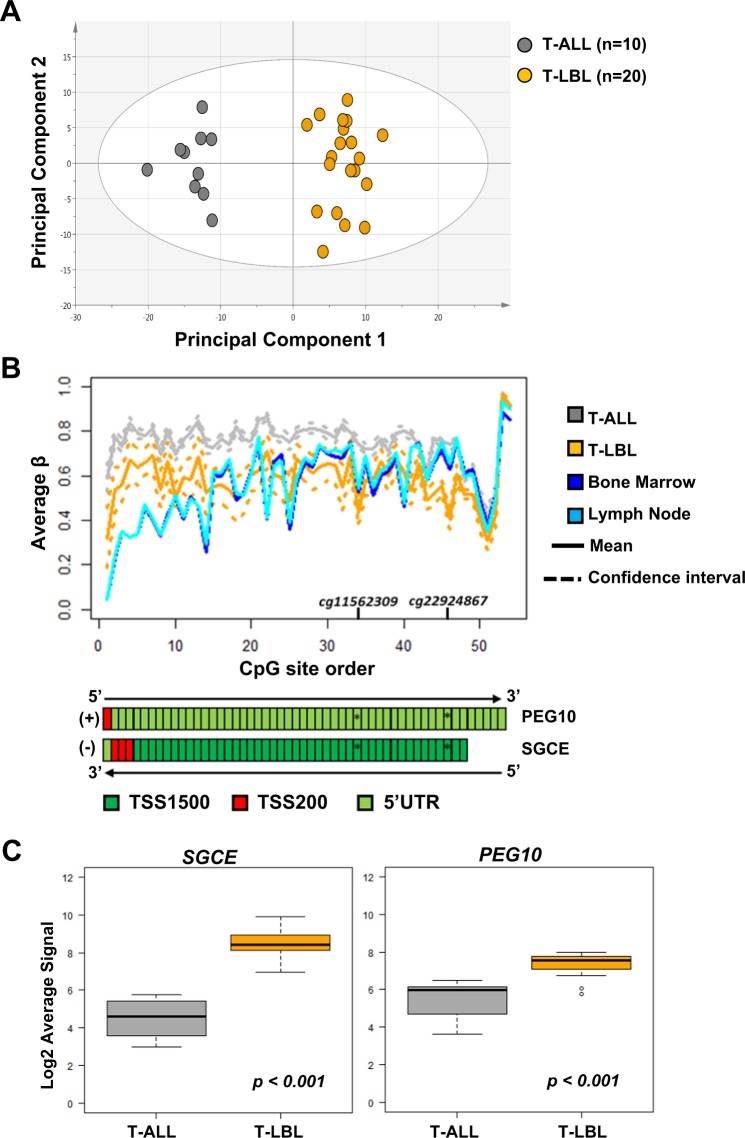
Integrated methylation and expression analysis of differentially methylated CpG sites between T-ALL and T-LBL. **a** PCA scoring plot showing separate clustering of Basso´s cohort of pediatric T-ALL (*n* = 10) and T-LBL (*n* = 20) samples based on log transformed average signal of 100 (219 probes) out of 110 unique genes corresponding to the 128 DM-CpGs. The gene expression data of T-ALL and T-LBL samples, analyzed by HG-U133Plus2.0 GeneChip arrays, was retrieved from the public data repository (GSE29986) (Basso, Mussolin et al. 2011). **b** Mean methylation levels at single CpG site resolution in *PEG10* (+sense strand) and *SGCE* (-antisense strand) promoter regions are compared between T-ALL (gray), T-LBL (yellow) and non-malignant lymph node (cyan) and bone marrow (blue) reference samples. The significant differentially methylated CpG sites are marked and the 95% confidence interval, based on the T distribution, is represented by dashed lines while the mean avg. β is represented as a solid line. **c** Boxplots comparing the gene expression of neighboring *SGCE* and *PEG10* genes in T-ALL (*n* = 10) and T-LBL (*n* = 20) patient cohort (Basso, Mussolin et al. 2011). The *p*-value is determined by Welch´s two sample *T*-test.

The potential functional relevance of the 128 DM-CpGs was evaluated by retrieving publicly available gene expression array data of T-ALL (*n* = 10) and T-LBL (*n* = 20)^5^. The public gene expression data^5^ was used to evaluate whether the transcriptomic profile of the genes, corresponding to the identified 128 DM-CpGs, also distinguished between T-ALL and T-LBL. The 128 DM-CpGs mapped to a total of 110 unique genes out of which gene expression data were available for 100 genes (219 gene expression probes) in 10 T-ALL and 20 T-LBL pediatric patients^5^. PCA of the log2-transformed average signal values of 219 gene expression probes clustered T-ALL and T-LBL samples separately (Fig. 4a). This showed that the differentially methylated CpG loci identified in our cohort of T-ALL and T-LBL patients, also separated the two diseases based on the corresponding gene expression profile in a separate cohort of T-ALL and T-LBL patients^5^ (Fig. 4a). Gene enrichment analysis of these 110 genes, revealed significant enrichment of transmembrane and membrane-associated proteins (Supplementary Table S1).

An integrated DNA methylation and gene expression analysis using Basso´s cohort was performed for the top ten most significant DM-CpGs (Supplementary Table S3). Amongst the top most significant DM-CpGs, two sites hypomethylated in T-LBL compared to T-ALL, mapped to the shared promoter region of *SGCE* and *PEG10* genes (Fig. 4b, Supplementary Table S3). The expression of the *SGCE* and *PEG10* genes was significantly differentially expressed in Basso's cohort (*SGCE* log2FC 3.98, *p* < 0.001 and *PEG10* log2FC 1.87, *p* < 0.001) with a higher gene expression in T-LBL than in T-ALL (Fig. 4c, Supplementary Table S3).

### Copy number variation analysis identified a higher frequency of Ch13q14.2 deletions in T-LBL

**Fig. 5:**
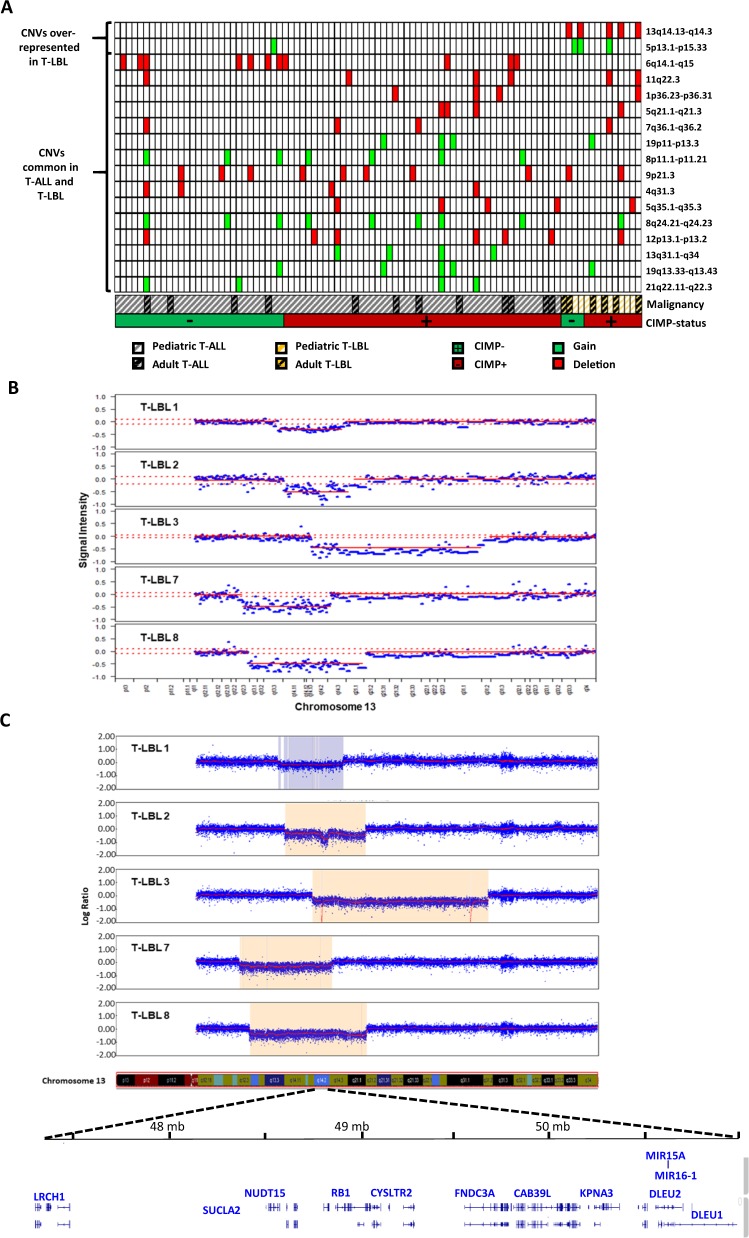
CNV analysis by HumanMethylation450K and CytoSNP-850K arrays. **a** Heat map showing copy number variations (deletions and gains) in T-ALL (*n* = 77) and T-LBL (*n* = 15) patients samples analyzed by HumanMethylation450K arrays. Samples are sorted as T-ALL and T-LBL and with increasing CIMP methylation percentage within the diagnoses. **b**–**c** Chromosome 13q14.2 deletions in 5 T-LBL patients analyzed by HumanMethylation450K arrays (**b**) and by Infinium CytoSNP-850K arrays (**c**). Interesting genes mapped within the minimal common region 13q14.2 are labeled.

To explore and compare the genomic landscape of T-ALL and T-LBL, we screened for recurrent deletions or gains in patient samples using the HumanMethylation450K arrays. A total of 17 chromosomal regions were identified with recurrent copy number variations out of which, a majority were common in both T-ALL and T-LBL patients (Fig. 5a, Table 2). Deletion in the CDKN2A/2B locus (9p21.3), a known recurrent deletion in T-ALL and T-LBL, was detected in 10% of T-ALL and 14% of T-LBL in our cohort (Table 2).

**Table 2:** Copy number variation (CNV) analysis by HumanMeth450K arrays.

Chr	Start	Stop	Ideogram CNV region	CNV type	T-ALL (*n* = 77)	T-LBL (*n* = 14)	Proportion with CNV T-ALL (%)	Proportion with CNV T-LBL (%)	*p-*value^a^
1	6,050,001	7,300,000	1p36.23-p36.31	Deletion	3	1	4	7	0.516
4	153,900 001	154,600 000	4q31.3	Deletion	4	0	5	0	1.000
5	10,001	40,650 000	5p13.1-p15.33	Gain	1	3	1	21	0.013*
5	100,250 001	106,300 000	5q21.1-q21.3	Deletion	3	1	4	7	0.516
5	170,750 001	180,905 260	5q35.1-q35.3	Deletion	4	1	5	7	1.000
6	80,250 001	88,400 000	6q14.1-q15	Deletion	11	0	14	0	0.201
7	148,850 001	153,550 000	7q36.1-q36.2	Deletion	3	1	4	7	0.516
8	40,800 001	43,838 887	8p11.1-p11.21	Gain	7	0	9	0	0.594
8	129,550 001	136,800 000	8q24.21-q24.23	Gain	8	1	10	7	1.000
9	21,400 001	23,850 000	9p21.3	Deletion	8	2	10	14	0.664
11	105,850 001	108,450 000	11q22.3	Deletion	4	2	5	14	0.252
12	12,500 001	14,100 000	12p13.1-p13.2	Deletion	6	1	8	7	1.000
13	46,950 001	51,500 000	13q14.13-q14.3	Deletion	0	5	0	36	<0.001*
13	84,450 001	115,109 878	13q31.1-q34	Gain	4	0	5	0	1.000
19	60,001	24,631 782	19p11-p13.3	Gain	3	1	4	7	0.516
19	48,500 001	59,118 983	19q13.33-q13.43	Gain	4	1	5	7	1.000
21	35,400 001	48,119 895	21q22.11-q22.3	Gain	4	0	5	0	1.000

^a^Fisher's exact test, *p-value < 0.05.

However, two CNVs, namely gain of 5p13.1-p15.33 (1 vs. 21%, *p* = 0.013) and deletion of 13q13.13-q14.3 (0 vs. 36%, *p* < 0.001), were significantly overrepresented in T-LBL samples as compared with T-ALL samples (Fig. 5a-b, Table 2). Focused analysis of the 13q14.13-q14.3 region identified 13q14.2 as the minor common genomic deletion region in the five T-LBL patients positive for the deletion. This region encompassed the tumor suppressor gene *RB1*, microRNA genes *MIR15A* and *MIR16-1*, as well as the *DLEU1* gene (Fig. 5c).

In order to validate the 13q14.2 deletions and gains in chromosome 5 in T-LBL, all fifteen T-LBL samples were further analyzed by CytoSNP-850K arrays (Table 3, Supplementary Table S4). SNP array analysis verified the gain of all or parts of chromosome 5 in three (21%) of the T-LBL samples (Supplementary Table S4). The recurrent 13q14.2 deletions in five (36%) of the T-LBL samples were also confirmed by SNP-array analysis (Table 3, Fig. 5c).

**Table 3:** Verification of CNV analysis by CytoSNP-850K v1.1 arrays.

CNV Ch 13q14.2 analysis							
Sample ID	HumMeth450K array			CytoSNP-850K v1.1 array			
	Start	End	Deletion (yes/no)	Start	End	Deletion (yes/no)	Genotype
T-LBL 1	38,900,001	54,850,000	Yes	40,021,077	46,802,657	Yes	Heterozygous
T-LBL 2	40,250,001	56,200,000	Yes	40,363,878	59,634,447	Yes	Heterozygous
				48,902,121	50,698,153	Yes	Homozygous
T-LBL 3	46,950,001	88,350,000	Yes	46,893,680	88,924,926	Yes	Heterozygous
				48,985,639	49,072,565	Yes	Homozygous
				84,559,815	84,705,359	Yes	Homozygous
T-LBL 4^a^	–	–	NA	–	–	No	–
T-LBL 5	–	–	No	–	–	No	–
T-LBL 6	–	–	No	–	–	No	–
T-LBL 7	30,400,001	51,500,000	Yes	29,499,142	51,21,608	Yes	Heterozygous
T-LBL 8	31,800,001	60,000,000	Yes	31,926,477	58,586,236	Yes	Heterozygous
T-LBL 9	–	–	No	–	–	No	–
T-LBL 10	–	–	No	–	–	No	–
T-LBL 11	–	–	No	–	–	No	–
T-LBL 12	–	–	No	–	–	No	–
T-LBL 13	–	–	No	–	–	No	–
T-LBL 14	–	–	No	–	–	No	–
T-LBL 15	–	–	No	–	–	No	–

^a^NA-inconclusive data.

## Discussion

The biology behind the different clinical manifestation in T-ALL and T-LBL is still not well established. Despite overlapping immunophenotypic and morphological features, T-ALL and T-LBL have divergent clinical manifestation, with lymph nodes/extramedullary tissue infiltration associated with T-LBL and a predominant bone marrow infiltration in T-ALL. By high-resolution genome-wide DNA methylation and copy number variation detection arrays, we aimed at exploring the methylomic and genomic landscape in T-ALL and T-LBL to identify molecular markers that could differentiate between the two diseases and could also help evaluate the biology behind the differential pattern of primary disease distribution. Neither global, nor genome-wide promoter-focused DNA methylation analysis by PCA, could separate T-ALL patients from T-LBL patients or distinguish between adult and pediatric patients. It has been shown previously that adult and pediatric T-ALL have significant overlap of leukemia-specific genetic and cytogenetic lesions^20,21^. It can therefore be speculated that adult and pediatric cases also have similar methylation profiles, as demonstrated by the co-clustering of adult and pediatric patients in our multivariate analysis. The largest variation in global and promoter-associated DNA methylation pattern between the patient samples was based on CIMP status, verifying the presence of epigenetic CIMP subgroups in a broader set of T-cell malignancies including pediatric and adult T-LBL and adult T-ALL patients, which has not been shown before^6,7,9^. Even though the prognostic significance of CIMP profiling in T-LBL and adult T-ALL could not be validated because of limited cohort size, the CIMP subgroups in aforementioned diagnoses showed similar differences in cellular proliferative history, as shown previously by us in pediatric T-ALL CIMP subgroups^9^. The CIMP+ T-LBL and adult T-ALL patients had older predicted epigenetic and mitotic age compared to the CIMP− patients, which is in line with our previously published data^9^.

In order to identify specific genomic loci with differential DNA methylation between T-ALL and T-LBL, we performed differential methylation analysis in the promoter-associated CpG sites, considering the functional relevance of DNA methylation in promoters. A set of 128 DM-CpG sites was identified, that separated T-ALL and T-LBL as distinct clusters, without reflecting methylomic variations based on tissue type, age, or CIMP heterogeneity. Functional analysis of genes corresponding to the 128 DM-CpGs between T-ALL and T-LBL revealed an overrepresentation of membrane and transmembrane associated proteins. Gene expression signatures, discriminating T-ALL and T-LBL identified in previous studies, were also shown enriched in genes encoding cellular adhesion proteins and extracellular matrix proteins^4,5^. This implies that T-ALL and T-LBL have biological differences that might govern the difference in their different disease manifestation. The most significant differentially methylated sites mapped to the shared CpG-island rich promoter region of Sarcoglycan-epsilon (*SGCE*) and its neighbor, paternally expressed gene 10 (*PEG10*), which are both maternally imprinted genes on chromosome 7q21^22^. PEG10 plays a vital role in placental formation and differentiation of adipocytes while *SGCE* gene encodes a transmembrane protein that links the actin cytoskeleton to extracellular matrix^23^. However, deregulated *PEG10* expression is associated with malignant transformation, affecting cell proliferation and apoptosis^24^. Both *PEG10* and *SGCE* are known to be overexpressed in high-risk B-cell chronic lymphocytic leukemia (B-CLL), with promoter DNA methylation regulating the gene expression^24^. Overexpression of *PEG10* has also been implicated in progression of various solid cancers including hepatocellular carcinoma^25^, pancreatic cancer^26^, breast cancer^27^ and prostate cancer^24^. Overexpression of *PEG10* in these solid tumors was shown to correlate with higher TNM stage and lymph node metastasis^28^. Interestingly, long non-coding RNA *PEG10*, also encoded from 7q21, was shown upregulated in diffuse large B cell lymphoma (DLBCL), which correlated with worse prognosis^29^. One of the oncogenic effects of *PEG10* in the solid cancers has been shown to promote metastatic migration and promoting epithelial-mesenchymal transition of neoplastic cells^24^. The overlapping epigenetic and transcriptomic disparities identified between T-ALL and T-LBL in our study might contribute to the different manifestations of the malignancies. However the functional relevance has to be further evaluated in order to conclude the epigenetic contribution to disease distribution.

Furthermore, we explored possible similarities and differences in genetic alterations between T-LBL and T-ALL. The most frequently commonly observed CNVs in T-ALL and T-LBL was the deletion of 9p21.3 including the CDKN2A/2B loci. Previous studies have also identified 9p21.3 deletions commonly recurrent in both T-ALL and T-LBL^2,30–32^.

Interestingly, we identified and validated a frequently deleted genomic region on chromosome 13q14.2 present in 36% (5/14) of the T-LBL samples, that was not observed in any T-ALL sample (0/77). Similarly, gain of all or parts of chromosome 5 were observed in T-LBL patients in our study cohort. Trisomy 5, gain of the whole chromosome 5, is recurrent in hematological malignant cases with hyperdiploid karyotype (chromosome number >50). Our findings are in line with a previous study that reported hyperdiploid karyotype significantly more frequent in T-LBL compared to T-ALL patients^33^.

The presence of 13q14.2 deletions in T-LBL samples has also been described in a previous study that reported 13q14.2 deletions in 2 out of 12 T-LBL (17%)^31^. However, that study also observed 13q14.2 deletions in 6 out of 57 (11%) T-ALL patients, which were not seen in our Nordic T-ALL cohort^31^. The 13q14.2 region partially overlaps with the most commonly deleted region in B-cell chronic lymphocytic leukemia (B-CLL)^34–36^ and contains *RB1, KCNRG, TRIM13, DLEU1/2, MIR16-1*, and *MIR15A* genes. In addition, 13q14 deletions have been observed in other hematological malignancies including mantle cell leukemia, multiple myeloma and acute myeloid leukemia^37–40^. One of the downstream inhibitory targets of *MIR16-1* and *MIR15A* include the anti-apoptotic *BCL2* gene. In B-CLL, loss of 13q14.2 region has been shown associated with reduced *MIR16-1* and *MIR15A* expression and increased *BCL2* mRNA levels^41^. Another study showed through Zebrafish modeling, that increased expression of *BCL2* favored the development of T-LBL over T-ALL^42^. However, further functional analyses are required to elucidate the link between 13q14.2 deletions, *BCL2* overexpression and T-LBL biology. If 13q14.2 deletions and subsequent *BCL2* overexpression are validated as potential oncogenic events in T-LBL, the next step would be to evaluate the efficacy of drugs like Venetoclax, for treating 13q14.2 deleted T-LBL patients. Venetoclax, a selective inhibitor of *BCL2*, is currently used as a targeted therapy for treating CLL^43^ and acute myeloid leukemia^44^, but its efficacy in treating T-LBL patients is yet to be determined.

In conclusion, we showed that DNA methylation CIMP subgroups, with prognostic significance in pediatric T-ALL, are also present in adult T-ALL, as well as in pediatric and adult T-LBL, suggesting a broader relevance of CIMP classification in T-cell malignancies. Furthermore, epigenetic and genetic profiling revealed molecular differences between T-ALL and T-LBL, which may contribute to the differential biology of the two neoplasms.

## Supplementary information

Supplementary Information
