# Supplementary material for: DNA methylation and copy number variation profiling of T-cell lymphoblastic leukemia and lymphoma
Source: Blood Cancer J. 2020 Apr 28;10(4):45. doi: 10.1038/s41408-020-0310-9 (PMC7188684; doi:10.1038/s41408-020-0310-9)
Supplement: Supplementary file 1 — Supplementary Information [file 41408_2020_310_MOESM1_ESM.pdf]

SUPPLEMENTARY INFORMATION

TABLE OF CONTENTS

Supplementary Tables……………………………………………………………………….…...1

Supplementary Table S1: ………………………………………..……...............1

Supplementary Table S2: ………………………………………..……...............2

Supplementary Table S3: …………………………...3

Supplementary Table S4: ………………………………………………………….....4

Supplementary Figures...…………………………………………………………….…...5

Supplementary Figure S1:………………………………………..……...............5

Supplementary Figure S2: ...……………………………………..……...............6

Supplementary Figure S3: ...…………………………...7

Supplementary Tables

Supplementary Table S1: Significantly enriched functional clusters (defined as Enrichment Score of >1.3) as determined by DAVID functional annotation clustering analysis of the 110 uniques genes implicated with 128 DM_CpGs.

Category	Term	PValue	Genes	Fold Enrichment
**Annotation Cluster 1 (Enrichment Score: 1.41)**				
GO:0005887	integral to plasma membrane	0.01	KCNJ15, PTPRD, CASR, LYN, GPER, NRXN3, ADORA2A, PCDHB2, DDR1, CD55, SGCE, CHRNA2, HTR2A	2.15
GO:0031226	intrinsic to plasma membrane	0.02	KCNJ15, PTPRD, CASR, LYN, GPER, NRXN3, ADORA2A, PCDHB2, DDR1, CD55, SGCE, CHRNA2, HTR2A	2.10
GO:0044459	plasma membrane part	0.02	KCNJ15, CASR, PTPRD, BBS7, LYN, ADORA2A, GPER, NRXN3, PCDHB2, FMN1, DDR1, CD55, LHFPL5, COLQ, RHEB, SGCE, PAK1, CHRNA2, HTR2A	1.70
UP_SEQ_FEATURE	topological domain:Extracellular	0-04	KCNJ15, CASR, PTPRD, LRRN4CL, ADORA2A, GPER, NRXN3, MRC2, GPR78, PCDHB2, OR10J5, OR9Q2, CSMD1, DDR1, CDH9, TNFRSF19, OR8B2, IL15RA, SGCE, MMD2, HTR2A, CHRNA2	1.55
GO:0005886	plasma membrane	0-04	KCNJ15, CASR, BBS7, ADORA2A, OR9Q2, CDH9, MCOLN3, TIAM1, PAK1, CHRNA2, PTPRD, LYN, NRXN3, GPER, GPR78, PCDHB2, OR10J5, FMN1, DDR1, CD55, LHFPL5, COLQ, OR8B2, RHEB, SGCE, NEU1, HTR2A	1.41
**Annotation Cluster 4 (Enrichment Score: 1.37)**				
GO:0016021	integral to membrane	0.01	KCNJ15, CASR, NDST4, ADORA2A, TOMM20L, LEMD1, CSMD1, OR9Q2, CDH9, MCOLN3, OTOP3, TNFRSF19, IL15RA, MMD2, CHRNA2, PTPRD, LRRN4CL, LYN, NRXN3, GPER, TMEM225, MRC2, GPR78, PCDHB2, OR10J5, KIAA1467, CPT1A, DDR1, CD55, LHFPL5, SLC25A31, OR8B2, HHLA2, SGCE, SLC18A1, SPNS1, EMP2, HTR2A	1.41
GO:0031224	intrinsic to membrane	0.01	KCNJ15, CASR, NDST4, ADORA2A, TOMM20L, LEMD1, CSMD1, OR9Q2, CDH9, MCOLN3, OTOP3, TNFRSF19, IL15RA, MMD2, CHRNA2, PTPRD, LRRN4CL, LYN, NRXN3, GPER, TMEM225, MRC2, GPR78, PCDHB2, OR10J5, KIAA1467, CPT1A, DDR1, CD55, LHFPL5, SLC25A31, OR8B2, HHLA2, SGCE, SLC18A1, SPNS1, EMP2, HTR2A	1.36
SP_PIR_KEYWORD	membrane	0.01	KCNJ15, CASR, NDST4, BBS7, ADORA2A, CYP11B2, TOMM20L, LEMD1, CSMD1, OR9Q2, CDH9, MCOLN3, OTOP3, TNFRSF19, IL15RA, PAFAH1B1, MMD2, CHRNA2, PTPRD, LRRN4CL, LYN, NRXN3, GPER, TMEM225, MRC2, GPR78, PCDHB2, OR10J5, CPT1A, KIAA1467, FMN1, DDR1, CD55, LHFPL5, SLC25A31, OR8B2, HHLA2, RHEB, NEU1, SGCE, SLC18A1, SPNS1, EMP2, HTR2A	1.35
UP_SEQ_FEATURE	transmembrane region	0.02	KCNJ15, CASR, NDST4, ADORA2A, TOMM20L, LEMD1, CSMD1, OR9Q2, CDH9, MCOLN3, OTOP3, TNFRSF19, IL15RA, MMD2, CHRNA2, PTPRD, LRRN4CL, NRXN3, GPER, TMEM225, MRC2, GPR78, PCDHB2, OR10J5, KIAA1467, CPT1A, DDR1, LHFPL5, SLC25A31, OR8B2, HHLA2, SGCE, SLC18A1, SPNS1, EMP2, HTR2A	1.40
SP_PIR_KEYWORD	transmembrane	0.02	KCNJ15, CASR, NDST4, ADORA2A, TOMM20L, LEMD1, CSMD1, OR9Q2, CDH9, MCOLN3, OTOP3, TNFRSF19, IL15RA, MMD2, CHRNA2, PTPRD, LRRN4CL, NRXN3, GPER, TMEM225, MRC2, GPR78, PCDHB2, OR10J5, KIAA1467, CPT1A, DDR1, LHFPL5, SLC25A31, OR8B2, HHLA2, SGCE, SLC18A1, SPNS1, EMP2, HTR2A	1.39
UP_SEQ_FEATURE	topological domain:Cytoplasmic	0.04	KCNJ15, CASR, NDST4, ADORA2A, TOMM20L, OR9Q2, CSMD1, CDH9, IL15RA, TNFRSF19, MMD2, CHRNA2, PTPRD, LRRN4CL, NRXN3, GPER, MRC2, GPR78, PCDHB2, OR10J5, CPT1A, DDR1, OR8B2, SGCE, SLC18A1, HTR2A	1.47
UP_SEQ_FEATURE	topological domain:Extracellular	0.04	KCNJ15, CASR, PTPRD, LRRN4CL, ADORA2A, GPER, NRXN3, MRC2, GPR78, PCDHB2, OR10J5, OR9Q2, CSMD1, DDR1, CDH9, TNFRSF19, OR8B2, IL15RA, SGCE, MMD2, HTR2A, CHRNA2	1.55

GO, Gene Ontology ; SP, Swiss-Prot ; PIR, Protein Information Resource ; UP, Uniprot ; SEQ, Sequence

Supplementary Table S2: Number of differentially methylated CpG sites between T-ALL and T-LBL patients analysed by ChAMP algorithm.

**Differential Methylation Analysis**	**Number of Samples**		**Number of differentially methylated CpG sites***
	**T-ALL**	**T-LBL**	
T-ALL vs. T-LBL	77	15	1987
Adult T-ALL vs. T-LBL	12	7	2848
Pediatric T-ALL vs. T-LBL	65	8	1728

**adj. pval <0.05, absΔβ >0.3*

Supplementary Table S3: Integrated analysis of differential methylation and differential expression of top ten most significant differentially methylated CpG sites from the 128 DM-CpGs signature. Log2 fold change (log2FC) and delta β of differentially methylated genes is compared, using Basso´s gene expression data of T-ALL (n=10) and T-LBL (n=20) samples (Basso, Mussolin et al. 2011) and methylation data from our patient cohort of T-ALL (n=77) and T-LBL (n=15).

	HumMeth450K Array						GEX Array (Basso *et al.*)				
**Gene**	**CpG ID**	**Feature**	**T-ALL Avg. β (Mean)**	**T-LBL Avg. β (Mean)**	**delta β**	**Adj. P-value**	**Probe ID**	**T-ALL Log2 Avg. Signal (Mean)**	**T-LBL Log2 Avg. Signal (Mean)**	**Log2FC**	**P-value**
SGCE/PEG10	cg22924867	TSS1500	0.74	0.4	-0.33	4.61E-23	204688_at (SGCE)	4.54	8.52	3.98	3.39E-8
							212092_at (PEG10)	5.47	7.33	1.87	1.00E-3
C10orf88	cg10477193	TSS1500	0.74	0.38	-0.36	6.17E-20	219240_s_at	8.52	8.99	0.47	2.69E-3
HECW2	cg00402533	TSS1500	0.82	0.40	-0.42	2.71E-18	232080_at	5.83	7.45	1.62	1.66E-6
PECI	cg09054876	TSS1500	0.02	0.38	0.36	8.02E-17	218025_s_at	11.29	10.42	-0.87	3.63E-5
SGCE/PEG10	cg11562309	TSS1500	0.74	0.43	-0.31	1.33E-16	204688_at (SGCE)	4.54	8.52	3.98	3.39E-8
							212092_at (PEG10)	5.47	7.33	1.87	1.00E-3
SPNS1	cg06743973	TSS1500	0.78	0.44	-0.34	1.57E-16	223173_at	9.87	9.53	-0.34	2.05E-1
PI15	cg24349665	TSS200	0.11	0.67	0.56	3.14E-16	229947_at	3.67	6.58	2.91	3.08E-4
LRRN4CL	cg15538427	5'UTR	0.76	0.42	-0.33	1.84E-15	1556427_s_at	6.17	7.87	1.70	1.01E-7
KIAA1467	cg25108548	TSS1500	0.74	0.35	-0.38	8.53E-14	1559302_at	5.96	6.79	0.83	3.85E-3
MYLK4	cg02404304	TSS1500	0.12	0.58	0.45	2.12E-13	1561503_at	4.75	6.58	1.84	2.17E-3

Log2FC, T-LBL log2 avg. signal - T-ALL log2 avg. signal; delta β, T-LBL avg. β - T-ALL avg. β; Adj. P-value, Bonferroni adjusted p-value; P-value, Welch´s two sample T-test.

Supplementary Table S4: Verification of gains of chromosome 5 in 15 T-LBL patients by CytoSNP-850K v1.1 arrays.

**CNV Ch 5 analysis**					
**Sample ID**	**HumMeth450K array**			**CytoSNP-850K v1.1 array**	
	**Start**	**End**	**Gain (yes/no)**	**CNV type**	**Gain (yes/no)**
T-LBL 1	-	-	no	-	no
T-LBL 2	10 001	180 905 260	yes	Trisomy 5	yes
T-LBL 3	-	-	no	-	no
T-LBL 4*	-	-	no	-	no
T-LBL 5	-	-	no	-	no
T-LBL 6	10 001	180 905 260	yes	Trisomy 5	yes
T-LBL 7	10 001	40 650 000	yes	Partial Trisomy 5p	yes
T-LBL 8	-	-	no	-	no
T-LBL 9	-	-	no	-	no
T-LBL 10	-	-	no	-	no
T-LBL 11	-	-	no	-	no
T-LBL 12	-	-	no	-	no
T-LBL 13	-	-	no	-	no
T-LBL 14	-	-	no	-	no
T-LBL 15	-	-	no	-	no

**NA- inconclusive data*

Supplementary Figures

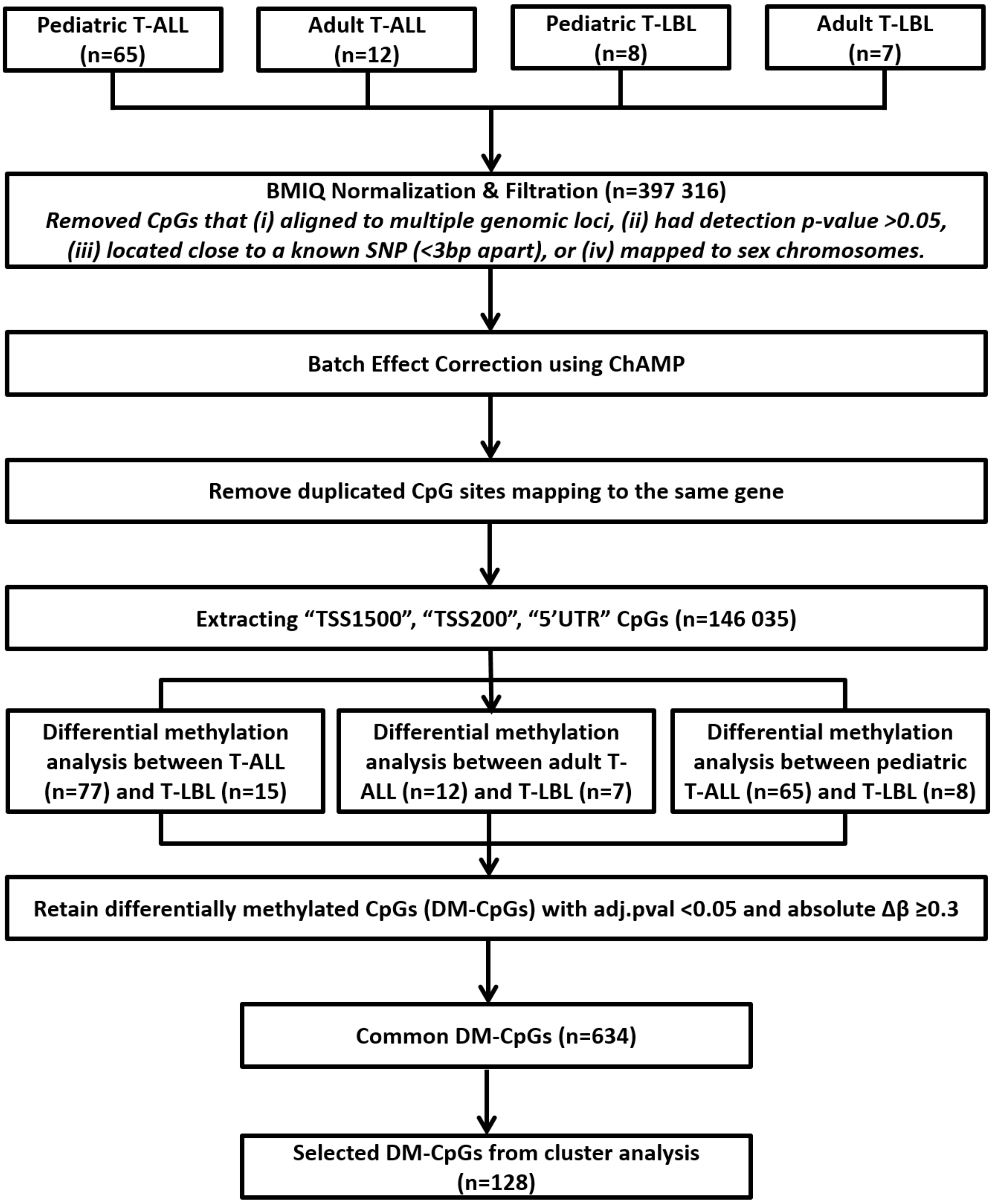

Supplementary Figure S1: Scheme for differential methylation analysis between T-ALL and T-LBL. Analysis was performed using ChAMP algorithm.

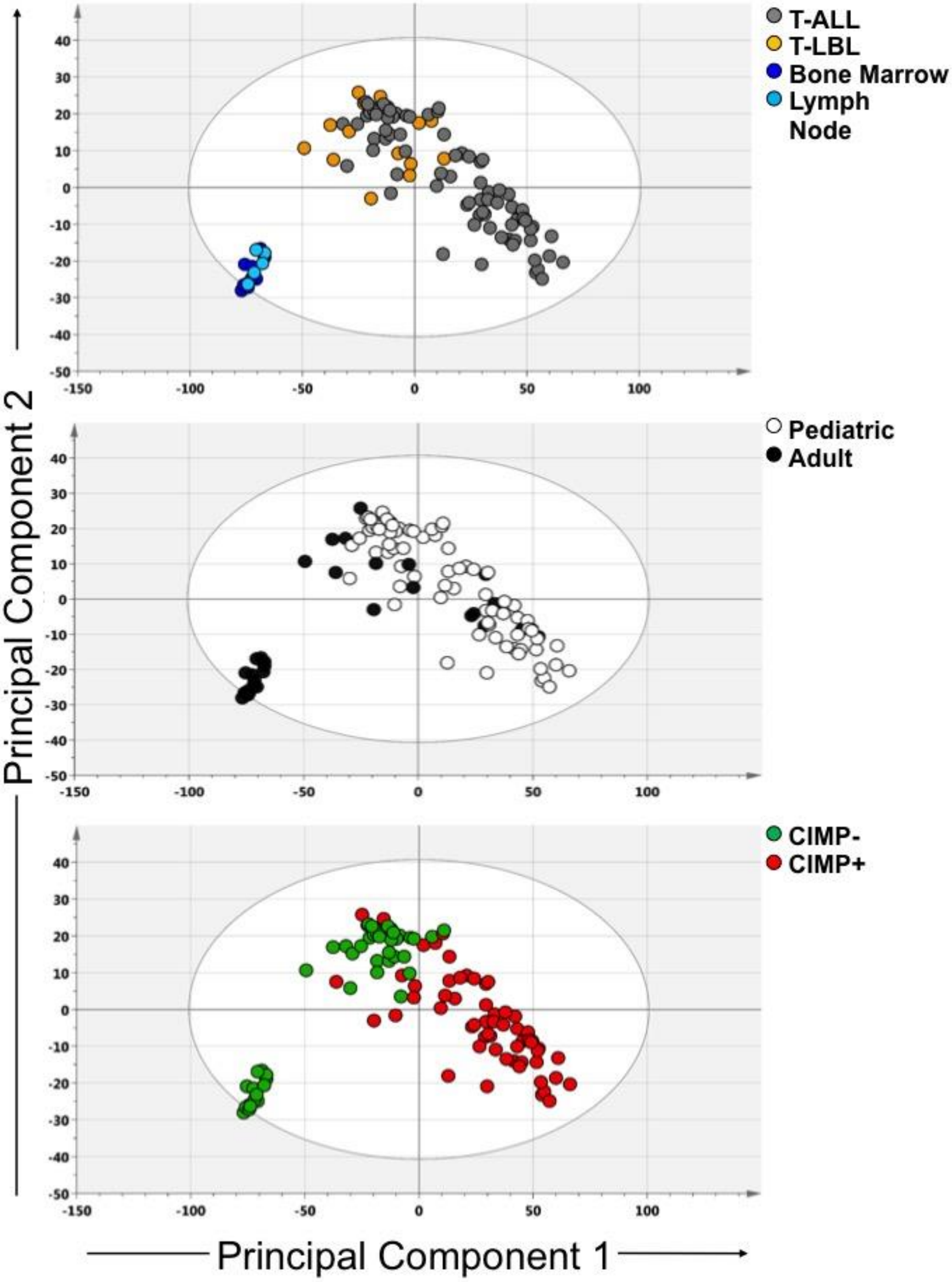

Supplementary Figure S2: Principal component analysis (PCA) scoring plots based on centrally scaled average β values of all promoter-associated CpGs (n= 146035). The samples in the PCA plots are colored by sample type, age group and by CIMP status.

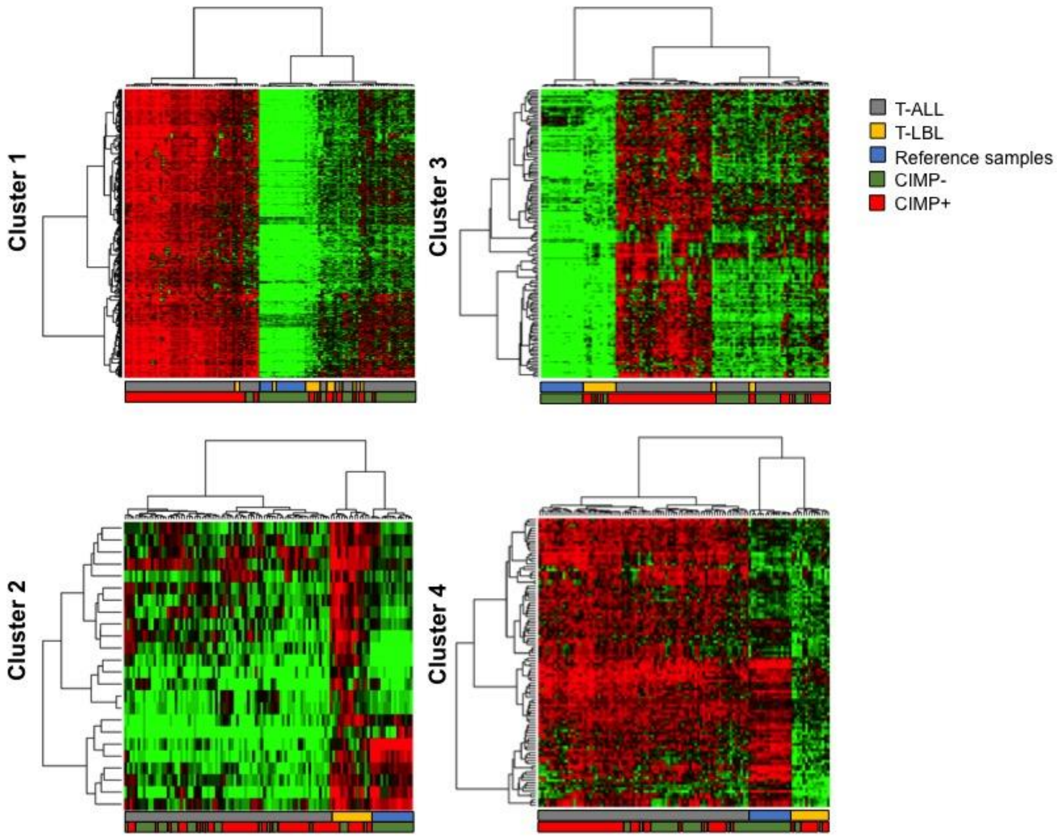

Supplementary Figure S3: Heat maps showing cluster analysis of CpGs in the four identified clusters (Figure 1), cluster 1 (n=367 CpGs), cluster 2 (n=24 CpGs), cluster 3 (n= 139 CpGs) and cluster 4 (n=104 CpGs). T), to identify clusters of DM-CpGs that show largest differences between T-ALL and T-LBL and not representing different CIMP profiles. CIMP subgroup and cell types are marked below the heat map.
